# Concurrent Howell–Jolly body‐like inclusions, Barr bodies and Döhle bodies in an immunosuppressed patient with sepsis

**DOI:** 10.1111/bjh.70591

**Published:** 2026-06-02

**Authors:** Vandana Panakkal, Nana P. Matsumoto

**Affiliations:** ^1^ Department of Pathology University of Pittsburgh School of Medicine Pittsburgh Pennsylvania USA

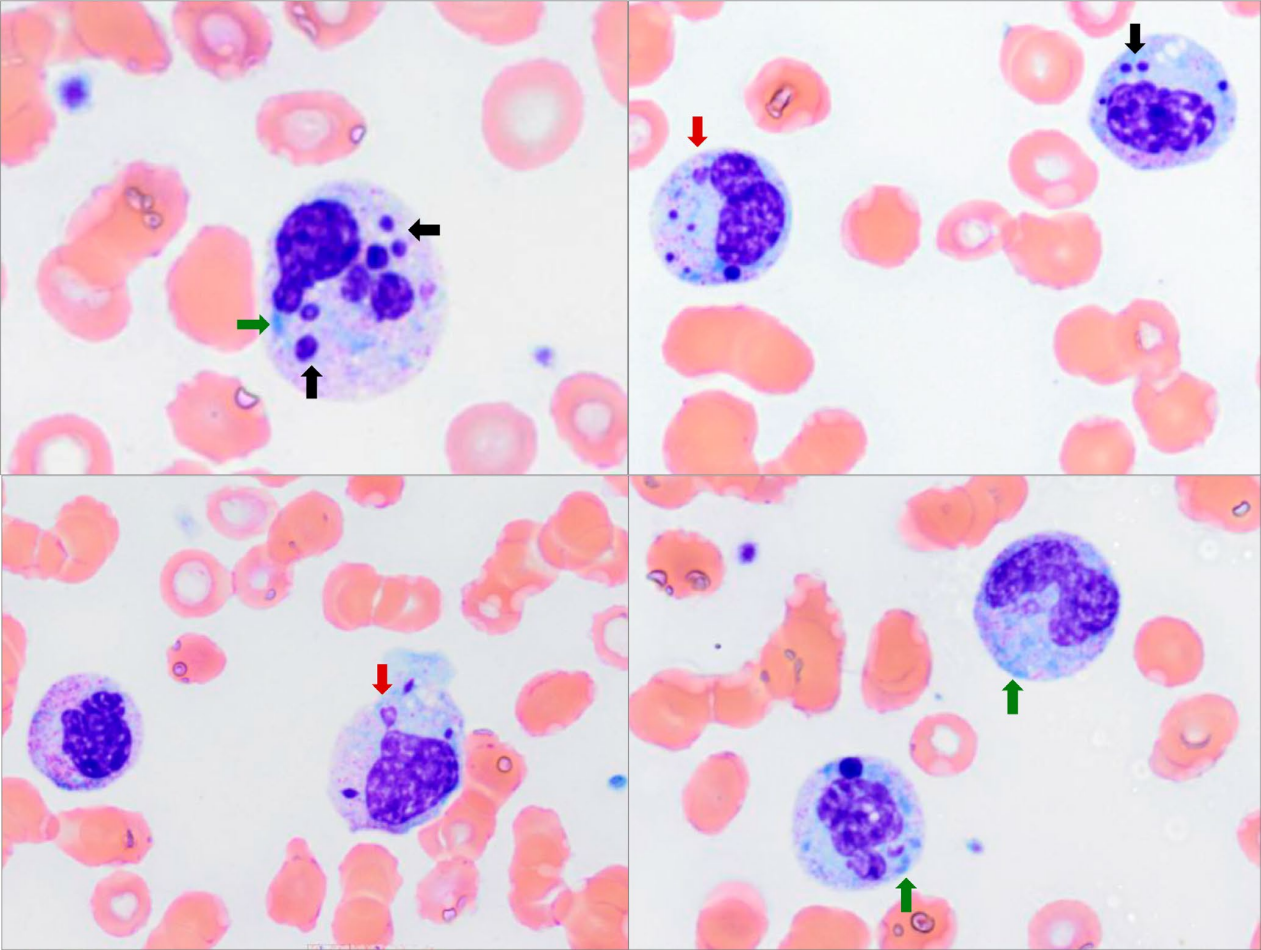

A 31‐year‐old woman with a history of DiGeorge syndrome, extra‐nodal marginal zone lymphoma of mucosa‐associated lymphoid tissue of the lung, allogeneic haematopoietic stem cell transplantation for T‐cell immunodeficiency and subsequent post‐transplant lymphoproliferative disorder (diffuse large B‐cell lymphoma) in remission, presented with gastrointestinal and upper respiratory symptoms. She had *Streptococcus pneumoniae* sepsis, acute multifocal pneumonia and viral gastroenteritis. Full blood count (FBC) showed anaemia (haemoglobin: 92 g/L, normal: 116–146 g/L), thrombocytopenia (platelet count: 102 × 10^9^/L, normal 150–450 × 10^9^/L) and normal white bloocd cell count of 8.8 × 10^9^/L (normal: 3.8–10.6 × 10^9^/L).

The peripheral blood film (all images, Wright's stain, objective x100) demonstrated mildly left‐shifted granulocytes with morphological atypia, including atypical lobation and uneven cytoplasmic granulation. There were multiple variably sized basophilic, globular cytoplasmic inclusions in neutrophils and monocytes, consistent with Howell–Jolly body‐like inclusions (black arrow) and numerous pale grey‐blue, oval‐to‐elongated cytoplasmic Döhle bodies (green arrow). Additionally, some neutrophils displayed small drumstick‐shaped nuclear appendages attached to the nuclear lobes, consistent with Barr bodies (red arrow).

Howell–Jolly body‐like inclusions are deoxyribonucleic acid (DNA)‐based cytoplasmic inclusions formed from fragmented nuclear material. They are typically seen in clinical scenarios such as immunosuppressive therapy, chemotherapy, myelodysplastic syndromes, human immunodeficiency virus (HIV), coronavirus disease 2019 (COVID‐19) and antiviral medication use. Morphologically, they resemble the basophilic inclusions of true Howell–Jolly bodies in erythrocytes.^1^, ^2^ In contrast, a Barr body is an inactivated X‐chromosome that appears as a small, hyperchromatic, drumstick‐shaped appendage attached to the neutrophil nucleus. This direct nuclear attachment helps distinguish Barr bodies from the fully cytoplasmic, detached Howell–Jollybody‐like inclusions.^1^, ^3^

Döhle bodies are pale blue‐grey cytoplasmic inclusions (1–2 μm) that represent strands of rough endoplasmic reticulum within neutrophils. They most commonly appear in the setting of accelerated granulopoiesis, including infections, inflammatory conditions and myeloid neoplasms. Giant Döhle bodies are one of the characteristic morphological features of May–Hegglin Anomaly. They may also be observed in monocytes and lymphocytes.^1^

Accurate identification of these structures is essential to prevent misinterpretation, as Howell–Jolly body‐like inclusions and Döhle bodies can mimic infectious morulae of *Anaplasma* or *Ehrlichia* species or myelodysplastic cytological abnormalities. Recognising these structures helps avoid unnecessary infectious evaluations and, when correlated with appropriate clinical contexts such as infection or physiological stress, reduces the risk of attributing reactive changes to dysplasia.

## PATIENT CONSENT STATEMENT

The images submitted were acquired as part of routine clinical care and are anonymized; hence, an informed consent is not obtained as it is not required as per institutional policy.
